# On the Role of the Patella, ACL and Joint Contact Forces in the Extension of the Knee

**DOI:** 10.1371/journal.pone.0115670

**Published:** 2014-12-23

**Authors:** Daniel J. Cleather, Dominic F. L. Southgate, Anthony M. J. Bull

**Affiliations:** 1 School of Sport, Health and Applied Sciences, St. Mary's University, Twickenham, United Kingdom; 2 Department of Bioengineering, Imperial College London, South Kensington Campus, London, United Kingdom; University of California Davis, United States of America

## Abstract

Traditional descriptions of the knee suggest that the function of the patella is to facilitate knee extension by increasing the moment arm of the quadriceps muscles. Through modelling and evidence from the literature it is shown in this paper that the presence of the patella makes the ability of the quadriceps to rotate the thigh greater than their ability to rotate the tibia. Furthermore, this difference increases as the knee is flexed, thus demonstrating a pattern that is consistent with many human movements. This paper also shows that the anterior cruciate ligament plays a previously unheralded role in extending the shank and that translation at the tibiofemoral and patellofemoral joints is important in improving the capacity for thigh rotation when the knee is flexed. This study provides new insights as to how the structure of the knee is adapted to its purpose and illustrates how the functional anatomy of the knee contributes to its extension function.

## Introduction

The way in which the human knee joint extends has received considerable attention and is described primarily as a function of the extension of the tibiofemoral joint by the action of the quadriceps (mediated by the patella). However, this is a joint-based description of patellar function, based upon the assumption that the joint acts as a single degree of freedom hinge between the segments, and that the muscles rotate the segments about this hinge. However, this fundamental assumption does not hold true for the knee, where the bony anatomy provides little restraint and there is no obvious structure that could be considered to act as a hinge (the principal restraint is provided through tethering by a limited number of ligaments [1]). Equally, the knee extensors differentially rotate the leg (thigh) and shank segments. Recently, segmental descriptions of movement have been proposed which describe the rotations of body segments rather than rotations of joints [2]–[6]. These are then used to consider how muscles, ligaments and joint reaction forces create rotation of the segments about their centre of mass (COM). The restraint provided by the passive structures of the joint is captured by considering their influence on the rotation of segments. Given the unique structure of the knee a segment-based approach can provide a clearer analysis of knee joint extension (as will be shown in this paper).

The role of the patella has been of particular interest to biomechanists. Despite early discussion [7], [8] as to even the existence of a functional role for the patella (and consequently the practice of patellectomy [9], [10]) there is now a fairly unanimous consensus that the function of the patella is to increase the effective moment arm of the patellar tendon (PT) about the tibiofemoral joint and to consequently magnify the extension moment produced by the quadriceps muscle group about the knee [7], [11]. Of course, this is based upon a joint-based analysis.

Early commentators on the function of the patella assumed that it acted as a smooth pulley, such that the force in the quadriceps tendon (QT) was matched by that in the PT throughout the movement of the knee [7], [12]–[14]. In reality, the patella functions as a lever, changing the position of its pivot point in order to maintain force and moment equilibrium about the patella [15]–[18] as it flexes and processes around the trochlea of the femur [11]. This characteristic movement of the patella results in some important changes in the geometry of the extensor apparatus of the knee. Firstly, the articulation of the patella in combination with the changing centre of rotation of the tibiofemoral joint produces a change in the moment arm of the patella tendon about the knee [7], [19], [20]. Secondly, the imperative to maintain force and moment equilibrium at the patella produces a changing relationship between the force in the QT and PT, a fact that has been demonstrated in both experimental [15]–[17] and modeling [18], [11] studies. In particular the ratio of PT to QT forces (P/Q ratio) changes from around 1∶1.1 at full extension to 1∶0.6 at 120° of knee flexion [21]. Thirdly, the movement of the patellofemoral and tibiofemoral joints changes the orientation of the PT, changing its resultant action from providing an anterior shear on the tibial plateau in early knee flexion, to a posterior shear at deeper knee flexion angles [17], [22]–[25].

Although these changes in geometry are known, the fundamental reasons for them have not been described, and the presence of the patella is normally justified by the effect it has on the moment arm of the QT about the tibiofemoral joint. It is the contention of this article, that this is a function of a joint-based analysis. In particular, this article will show that the presence of the patella allows the quadriceps muscle group to exert a different rotation effect on the tibial and femoral segments, an effect that cannot be captured underneath the typical assumptions of a joint-based analysis. The difference in the rotation of the tibial and femoral segments is important in properly understanding the function of the extensor apparatus of the knee.

## Materials and Methods

In this study, a simple two dimensional sagittal plane model based upon existing data sets [24], [26], [27] is employed to explore the extension function of the lower limb. The purpose of the model is to describe the effective geometry of the extensor mechanism of the quadriceps – and in particular to describe the relative tendency of tension in the vastus parts of the quadriceps to create rotation of the tibial and femoral segments. This is achieved by determining all of the forces that act upon the tibial, femoral and patellar segments that are created by tension in the quadriceps and then by calculating the tendency of each of these forces to create rotation of the segments about their COM. It should be noted that the scope of this study is only to consider the relative moment generating capacity of the muscles and ligaments at the knee.

The model consists of three rigid linked segments with zero mass representing the femur, tibia and patella. Flexion of the knee is simulated by rotating the femur about a stationary tibia. At each flexion angle the model is assumed to be in static force and moment equilibrium (i.e. each segment is in static force and moment equilibrium), and the only forces acting upon each segment are those that arise due to tension in the quadriceps. Therefore the resultant force acting on each segment is zero (note that because the resultant force acting upon each segment is zero, that the moment acting upon each segment is independent of the reference point from which it is calculated). The tendency of tension in the quadriceps to create rotation of the tibial and femoral segments is determined by calculating the external moment that must be applied to the COM of each segment to maintain its static moment equilibrium. As rectus femoris is both a biarticular muscle, and creates a joint reaction force at the hip, this model is restricted to an analysis of the vastus parts of the quadriceps – i.e. those that only cross the knee.

**Figure 1:**
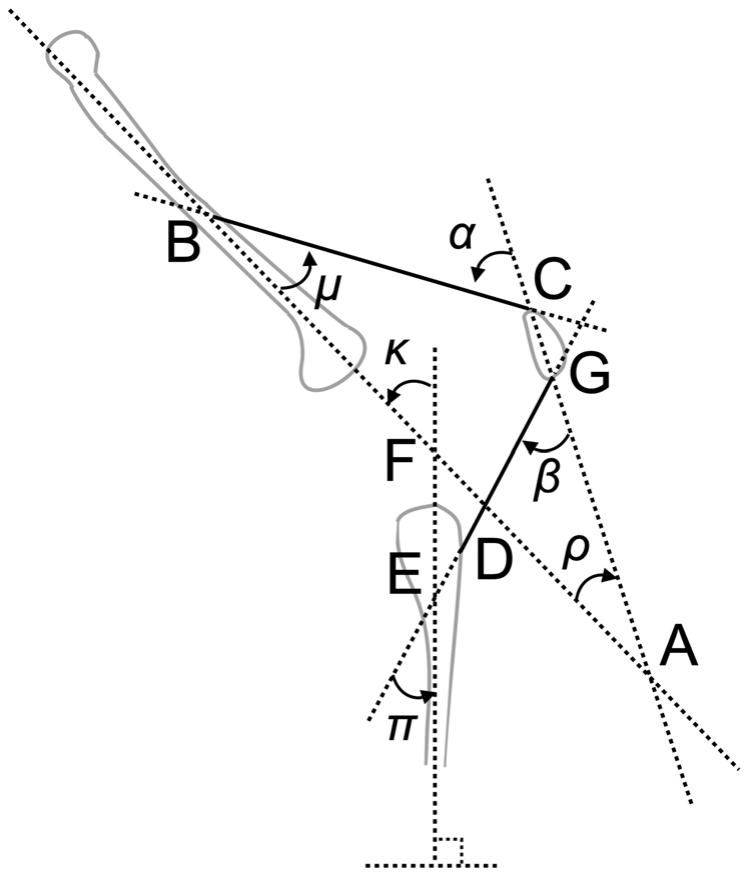
The geometry of the extensor mechanism of the knee (as described in this model). *κ*  =  knee flexion angle; *π*  =  patellar tendon angle (relative to the longitudinal axis of the tibia); *ρ*  =  patella tilt angle (relative to the longitudinal axis of the femur); *µ*  =  angle of the quadriceps relative to the femur; *α*  =  angle of incidence of the quadriceps tendon on the patella; *β*  =  angle of incidence of the patellar tendon on the patella.

The geometry of the model (as depicted in Fig. 1) is determined as follows. Firstly, the lines of action of all forces on the tibial, patellar, and femoral segments that solely arise from tension in the quadriceps muscle group are calculated at intervals of 5 degrees from full extension to 120 degrees of knee flexion (*κ*). The first step of this process is to calculate the lines of action of all muscle-tendon units. Initially, the regression equations of Herzog and Read [24] are used to calculate the angle between the longitudinal axis of the tibial segment and the PT (*π*), anterior cruciate ligament (ACL) and posterior cruciate ligament (PCL; the equations of Herzog and Read simply specify the relationship between knee flexion angle and the PT, ACL and PCL angles). Next, the data of Nha and colleagues [26] is used to determine the inclination of the patella relative to the femoral segment (*ρ*) for each interval (Nha and colleagues reported the inclination of the patella through a range of knee flexion angles. In the model described here, this data is then used to find a function representing the inclination of the patella for any given knee flexion angle). The line of action of the QT relative to the femoral segment (*µ*) is assumed to be constant up until the point at which the QT wraps around the distal femur and is taken from the anatomical data of Klein Horsman and colleagues [27] (the data set of Klein Horsman and colleagues describes the geometry of the lower limb in terms of 3D anatomical locations, and so it is a trivial task to calculate this angle).

Given the known angles described above (*κ*, *π*, *ρ*, *µ*) it is then possible to calculate the angles of incidence of the QT (*α*) and PT (*β*) on the patella from geometrical considerations. These relationships are outlined in Fig. 1. Firstly, consideration of the large triangle ΔABC, and given that *ρ* and *µ* are known, allows the calculation of the angle of incidence of the QT on the patella (*α*). Next, consideration of the small triangle ΔDEF, and given that *κ* and *π* are known, allows the determination of the angle ∠EDF. Finally, consideration of the triangle ΔADG, and given that *ρ* and ∠EDF are known, allows the calculation of the angle of incidence of the PT on the patella (*β*). Of course, this analysis only applies for one particular geometrical configuration of the knee, but it is a trivial task to repeat the analysis for other positions.

In this model, the patella is assumed to be in force and moment equilibrium at all knee flexion angles. Consequently, this produces a changing ratio of PT to QT forces (P/Q ratio) as the knee flexes, as has been described by previous authors. This ratio can be simply calculated from Equation 1, based upon the angles of incidence of the QT (*α*) and PT (*β*) on the patella. The P/Q ratio produced by this procedure is compared to previous research [18], [21] and the initial angle of the patella relative to the femur adjusted to provide the best possible fit. This is assumed to be a valid assumption as the data of Nha and colleagues [26] simply describes the relative change in patella tilt with increasing knee flexion, and does not determine the effective functional axis of the patella.
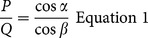



**Figure 2:**
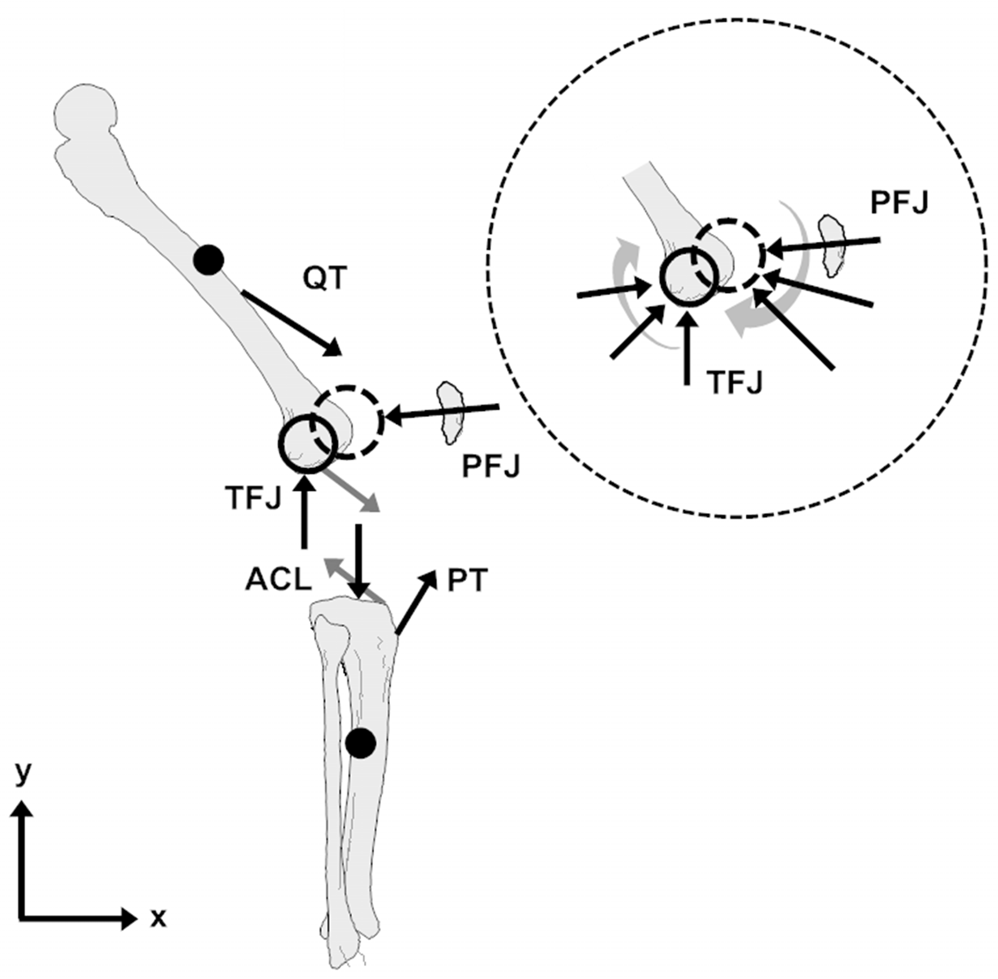
Rotation of femoral and tibial segments about their centre of masses. Forces acting on the segments include the quadriceps tendon force (QT), patellofemoral joint contact force (PFJ), tibiofemoral joint contact force (TFJ), patellar tendon force (PT) and cruciate ligament forces (either anterior cruciate ligament (ACL) or posterior cruciate ligament (PCL) force – the ACL is depicted here). The insert shows the changing point of application of the PFJ and TFJ on the femoral segment with increasing knee flexion.

Next, all of the forces that act upon the tibial, femoral and patellar segments as a result of tension in the quadriceps are calculated, by assuming a nominal tension of 1 N in the QT. Firstly, the tension in the PT is calculated based upon the P/Q ratio, giving the PT force acting on the tibia. The tension in the cruciate ligaments can then be calculated by assuming that they are the sole restraints to anterior/posterior shear of the tibia and using the cruciate ligament angles calculated earlier (note that this assumption means that only one of the cruciate ligaments is recruited at any joint angle). The final force acting on the tibia is the tibiofemoral joint contact force (TFJ) which is equal and opposite to the sum of the PT and cruciate ligament forces, and in this model is assumed to be directed through the COM of the tibia. Next the three forces acting upon the patella are calculated, based upon the assumption that the contact force between patella and femur maintains force equilibrium at the patella, by equilibrating the QT and PT forces. Finally, four forces act upon the femur; the QT force and the patellofemoral joint contact force (PFJ) which are assumed to be equal and opposite to the analogous forces acting upon the patella, and the TFJ and cruciate ligament forces which are equal and opposite to those acting on the tibia. The forces acting upon all three segments are depicted on Fig. 2.

The forces acting upon the femur and tibia are assumed to create rotation about the COM of each segment, the position of which is taken from the data of Klein Horsman and colleagues [27]. In addition, the origin of the QT on the femur, the insertion of the PT on the tibia and the origins and insertions of the cruciate ligaments are also taken from this data set. The point of application of the TFJ on the tibia is assumed to remain fixed throughout knee flexion however, the point of application of the TFJ on the femur changes during knee flexion. This is achieved by modelling the distal articular surface of the femur as a circle, and by assuming that flexion of the tibiofemoral joint is achieved by the combination of the femur rolling and sliding on the tibial plateau. Similarly the contact point of the PFJ with the femur is modelled by assuming that the patella articulates with a circle representing the trochlea. The QT is also assumed to wrap around this shape from 85 degrees of knee flexion. All of the geometry described above is also derived from the geometrical data of Klein Horsman and colleagues [27].

At this point, all of the force vectors acting on both femoral and tibial segments arising from 1 N of tension in the QT are known in conjunction with their effective point of application. Thus the moment created by each force for each 1 N of tension about the COM of the relevant segment can be calculated. It should be noted that as this calculation yields the moment per Newton of tension in the QT, the quantity calculated is simply a distance. For this reason, the results presented below describe the effective moment arm (in cm) of the rotation effect created by each structure that arises due to 1 N of quadriceps tension. That is, the actual moment exerted by a given structure, given a particular amount of quadriceps tension can be found by multiplying the effective moment arm of the structure by the amount of quadriceps tension. Equally, the net moment of all of the forces acting on each of the femoral and tibial segments that arises due to quadriceps tension would be calculated by multiplying the amount of quadriceps tension by the combined effective moment arm for the segment. In all of the figures presented below, a positive effective moment arm indicates a moment that would tend to extend the knee joint. Thus although the tibial and femoral segments rotate in opposite directions during knee extension in this paper both moments are presented as positive knee joint moments.

## Results

**Figure 3:**
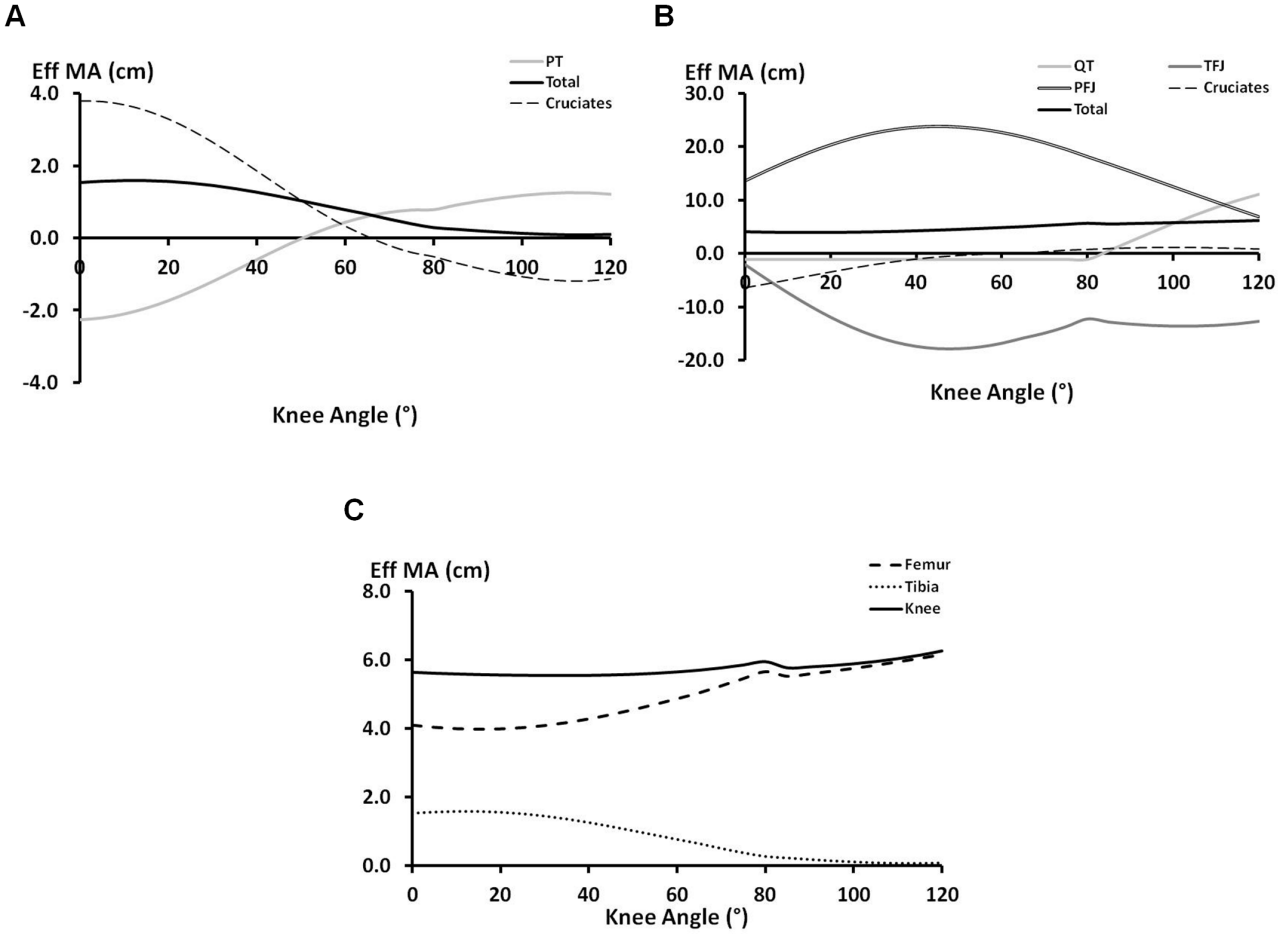
Effective moment arms (MA) of the forces acting upon the tibial and femoral segments that arise due to quadriceps tension; A) tibial segment; B) femoral segment; and C) combined effect on the knee.

The results of this modelling study suggest that at low angles of knee flexion, extension of the tibia is a consequence of the ACL force, as the PT actually impresses a flexion moment on the segment (Fig. 3A). At higher angles of knee flexion, the changing orientation of the PT allows it to directly contribute to tibial extension but a small flexion moment is imposed by the PCL. The combined effect of the cruciate ligament and PT forces produces the strongest extension moment in low angles of knee flexion. The extension moment acting on the tibial segment then diminishes until at a knee flexion angle of 80 degrees it is almost zero.

The rotation effects of the individual forces acting upon the femoral segment are of a greater magnitude than those acting on the tibia (Fig. 3B). However, in combination they produce an extension moment that is only about double that acting on the tibial segment (at least in full extension). It is clear that the influence of the PFJ is of critical importance in producing the extension effect, although the QT does become more important after it wraps around the femoral condyles. In contrast to the tibial segment, the extension moment acting on the femoral segment increases during knee flexion.

The sum of the extension moments acting upon the tibial and femoral segments gives the effective moment that extends the tibiofemoral joint (Fig. 3C). It is clear that the effective knee moment remains relatively constant during knee flexion however the way in which this knee moment is produced changes markedly. In particular, in early knee flexion there is an extension moment acting upon both the tibial and femoral segments however, in later knee flexion there is a greater contribution from femoral rotation.

## Discussion

Traditional joint-based descriptions of the patella suggest that its function is to act as a “joint spacer”, increasing the moment arm of the PT about the tibiofemoral joint. This traditional analysis also suggests that the moment arm of the PT changes as the knee flexes, first increasing as the knee flexes (from full extension) until it peaks at between 30 and 60 degrees of knee flexion, before then decreasing again (see Tsaopoulos and colleagues [19] for a review). It is also common for a joint-based analysis to include the assumption that the moment impressed by the PT on the tibial segment is equal and opposite to the moment impressed on the femoral segment.

The results of this study suggest a very different picture of patellar function. In particular, the segment-based analysis presented here suggests that tension in the quadriceps creates a different rotation effect on the tibial and femoral segments. This finding is one that is precluded by a traditional joint-based analysis due to the common assumption that the rotation effect on the tibial segment is equal and opposite to that on the femoral segment. Equally, this study suggests that the overall tendency of quadriceps tension to create rotation of the knee joint remains fairly constant throughout knee flexion, again in contrast to the joint-based analysis. Thus, the results of a segment-based analysis suggest that the role of the patella is to keep the effective moment arm of quadriceps tension about the knee joint constant, but also changing the way in which extension is achieved. In particular, with increasing angles of knee flexion, the tendency for the mechanism to extend the tibial segment is reduced whilst the tendency for femoral extension is increased. This pattern is consistent with the kinematics of a number of common movement patterns that involve closed kinetic chain flexion and extension of the knee through a full range of motion (e.g. squatting or lunging). During these activities at deeper knee flexion angles the extension of the lower limb is primarily achieved by the rotation of the thigh about a relatively stationary shank. It is only at shallower knee flexion angles where there is appreciable rotation of the tibial segment.

A further advantage of the segment-based analysis is inherent in the increased detail of the approach. For instance, underneath a joint-based analysis some of the detail relating to the function of the joint is lost, as the forces and structures that create the rotation are not explicitly modelled. This study therefore is able to provide further insight into the way in which quadriceps tension creates extension of the lower limb.

**Figure 4:**
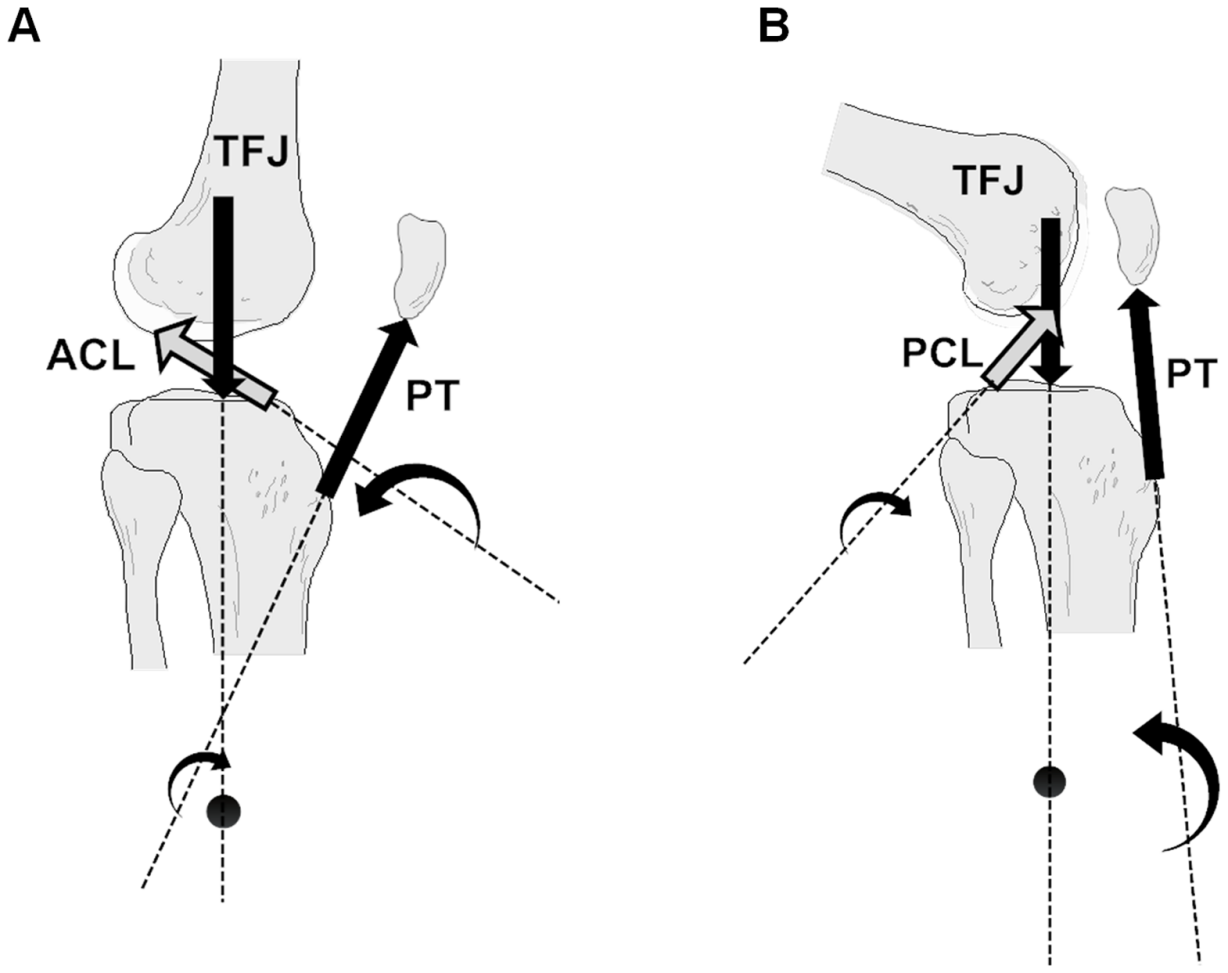
Extension of the tibia by quadriceps tension. A) when the knee angle is small the quadriceps actually exerts a flexion moment on the tibia and the extension moment is provided by the ACL; and B) with increased knee flexion angle, the patellar tendon can now produce an extension moment of the tibia, conversely recruitment of the PCL produces a flexion moment.

In Fig. 3A it was shown that in low angles of knee flexion the production of a tibial extension moment is due to the ACL force. This is entirely consistent with previous research exploring the geometry of the PT during knee flexion. At full extension the PT is orientated anteriorly relative to the tibial plane [22], [24], [27] such that it produces a flexion moment upon the tibial segment (see Fig. 4A). Thus the only way in which the PT can contribute to tibial extension is by creating an anterior shearing of the tibia, recruiting the ACL, which in turn exerts an extension moment on the tibia. This relationship is captured by a joint-based analysis (within the assumption of a hinge joint), but the detail is lost. In particular, a joint-based analysis might lead to the understanding that the role of the ACL is simply to resist anterior shearing of the tibia, neglecting its important function in extending the tibia. The contention that extension of the tibia requires tension in the ACL is also supported by studies that have demonstrated that the ACL is recruited at knee angles from around 0° to 50° during open chain knee extension activities [28] and even that increased extension moments increase the recruitment of the ACL [29].

At deeper knee flexion angles the PT is orientated more posteriorly and able to independently produce an extension of the tibia. However, this study (Fig. 3A) suggests that this is accompanied by a flexion moment produced by recruitment of the PCL (Fig. 4B). This is consistent with musculoskeletal modelling studies that have suggested increased recruitment of the PCL when the knee is more flexed (e.g., the work of Escamilla et al. [30]).

An understanding as to the rotation of the femoral segment (Fig. 3B) is more intuitive. The TFJ and cruciate ligaments create flexion moments (although the PCL has a mild extension effect in deeper knee flexion) whereas the PFJ creates an extension effect (that does vary with knee angle). The line of action of the QT passes close to the COM of the femur (prior to the wrapping of the QT about the femoral condyles) thus its extension ability is limited, but after wrapping, it is well placed to create an extension effect. Overall, the combination of these four forces creates a moment that extends the femoral segment that varies based on the changing positions at which they act, as well as their relative magnitudes. It is important to note that the changing position of the TFJ with increasing knee flexion contributes to the decreased flexion moment it exerts whereas the extension effect of the PFJ is increased by the movement of the patella. This demonstrates the importance of joint translation (the relative change in position of the joint surfaces caused by the relative translation of the two segments) at both the tibiofemoral and patellofemoral joints to the production of knee extension.

The mechanical understanding of knee joint function reported in this study is based upon a simplified model of the knee. In common with previous similar studies of knee joint function [23], [31], [32] this model is restricted to the sagittal plane, an assumption that is justified by the focus on only the extension behaviour of the knee. In addition, the anterior/posterior articular restraint at the knee joint is solely represented by the cruciate ligaments, and other structures of the knee (including the articular geometry and the menisci) are not considered. This simplification is justified in part by the fact that these structures are not likely to contribute significantly to anterior/posterior stability at the knee [1], [33] and in part by the fact that the model used here has been kept as simple as possible by design. In any case, the effect of the cruciate ligaments reported here can be taken as representing the upper bound of their potential role. It should also be noted that the effective moment arms of the individual forces are dependent on the choice of reference point (but the effective moment arm of the sum of the forces acting on a segment is invariant with respect to reference point as described in the methods).

The traditional conception as to the role of the patella is that it acts as a “spacer” to increase the moment arm of the PT about the tibiofemoral joint. This study suggests that the reason for the unique structure of the extensor mechanism of the lower limb is to increase the tendency of quadriceps tension to create femoral rotation as the knee flexion angle increases with a concomitant reduction in tibial rotation. This is important to adjust to the demands of many human locomotor tasks. In addition, this paper demonstrates that recruitment of the ACL is important to extension of the tibia, and similarly that the translations at the tibiofemoral and patellofemoral joints play a pivotal role in producing a strong extension of the femur.
